# Differences in amino acid digestibility between young and older adults: a randomized crossover study using the dual tracer method

**DOI:** 10.1016/j.ajcnut.2026.101361

**Published:** 2026-05-19

**Authors:** Fenna Hinssen, Romain Tessier, Dewi van Harskamp, Thom Huppertz, Marco Mensink, Nikkie van der Wielen

**Affiliations:** 1 Division of Human Nutrition and Health, Nutritional Biology, Wageningen University & Research, Wageningen, The Netherlands; 2 Amsterdam UMC, Department of Laboratory Medicine, Core Facility Metabolomics, Laboratory Genetic Metabolic Disease, University of Amsterdam, Amsterdam, The Netherlands; 3 Amsterdam UMC, Emma Center for Personalized Medicine, Amsterdam, The Netherlands; 4 Amsterdam UMC, Amsterdam Gastroenterology, Endocrinology & Metabolism, Amsterdam, The Netherlands; 5 FrieslandCampina, Amersfoort, The Netherlands; 6 School of Food & Nutritional Sciences, University College Cork, Cork, Ireland; 7 Animal Nutrition Group, Wageningen University & Research, Wageningen, The Netherlands

**Keywords:** protein, amino acid, aging, digestibility, dual tracer, stable isotope, older adults

## Abstract

**Background:**

Older adults may have reduced amino acid (AA) digestibility due to physiological changes in the digestive tract, but quantification of reduced AA digestibility in humans is lacking.

**Objectives:**

This randomized crossover study investigated differences in indispensable AA digestibility, primarily focusing on threonine and lysine, between young and older adults for milk, sorghum, and black beans using the dual tracer method.

**Methods:**

Ten young (21.8 ± 1.7 y) and 10 older (72.8 ± 3.8 y) adults ingested 20 g ^2^H-labeled protein from either milk, sorghum, or black beans, mixed with 400 mg of a ^13^C-labeled AA mixture, in a plateau feeding protocol on 3 separate test days. Blood was sampled before and at regular intervals over an 8-h period after meal consumption. The ^2^H- and ^13^C- enrichments of AAs in plasma samples were determined at steady state (5.5–8.0 h). The ratio of the ^2^H/^13^C-ratio between blood plasma and test meals was calculated. For lysine and threonine, this ratio was compared statistically between age groups and protein sources using linear mixed model analysis.

**Results:**

The isotope plasma-to-meal ratio for lysine and threonine was 19% lower in older adults compared with young adults for sorghum (*P* = 0.041), whereas it was not significantly different for milk (*P* = 0.277) and black beans (*P* = 0.849).

**Conclusions:**

Lysine and threonine digestion might be lower in older compared with young adults, but the effect differed among protein sources. Furthermore, research on how aging affects protein digestibility across protein sources is needed to optimize dietary protein recommendations for older adults.

This study was registered at clinicaltrials.gov as NCT05534464.

## Introduction

An increased protein intake of 1.2 g/kg body weight/day is advised for adults over the age of 65 years to maintain muscle mass [[1], [2], [3]] and support bone mineral density [4]. The ability to meet these protein and amino acid (AA) requirements with ingested foods depends on their nitrogen content, AA composition, and AA digestibility [5,6]. AA digestibility refers to the percentage of the dietary AAs that is digested and absorbed from the digestive tract [7].

As reviewed previously [8], several digestive organs undergo functional changes with increasing age. The mastication process changes, gastric emptying is delayed, and the production of pancreatic enzymes is decreased. These age-related changes may reduce protein digestion, which can have implications for dietary requirements in older adults. Although a reduction in protein digestion is expected, there is little direct evidence from in vivo studies in humans [8]. A meta-analysis of postprandial studies in humans showed that 45% of dietary protein-derived phenylalanine (Phe) appeared in the circulation in older adults, compared with 51% in young adults, and attributed these differences to aging-induced changes in protein digestion, absorption kinetics, splanchnic metabolism, and subsequent plasma availability [9]. Furthermore, splanchnic extraction was reported to be 2 times higher for leucine (Leu) [10] and 1.6 times higher for Phe [11] in older adults compared with younger adults. In addition, lower fecal protein digestibility in older (20 mo) rats compared with young (5 wk) rats was reported with a greater difference (≤17%) for protein sources that contained higher amounts of antinutritional factors (ANFs) [12].

On the basis of these observations, we hypothesized that AA digestibility is reduced in older adults compared with young adults and that this effect could differ among protein sources. Ideally, true ileal digestibility is measured using an oro-ileal balance method, but the terminal ileum is difficult to access in humans [7]. Alternatively, a dual stable isotope tracer method was developed with the aim to indirectly and less invasively measure protein digestion using an intrinsically labeled test protein and a reference protein intrinsically labeled with a different stable isotope. The isotope plasma-to-meal ratio derived from this method is then used as a measure of AA digestibility of the test protein relative to an AA mixture [13,14].

The dual tracer method relies on several assumptions, and there is a need to validate the method formally to accurately provide true ileal digestibility coefficients for protein sources [[15], [16], [17], [18]]. Nevertheless, its measured isotope plasma-to-meal ratios are suitable to identify differences in digestible AA quantities [19]. The dual tracer method encompasses the process of digestion and absorption but also includes some processes in splanchnic metabolism. Therefore, the measurement provides valuable information about the digestive handling of AAs in protein sources and can be used to compare young and older adults. However, when the isotopes are at labile positions, this can result in loss of label through exchange reactions in the postabsorptive splanchnic metabolism, which can impact outcomes.

Our study applied the dual tracer method with the aim to assess differences in AA digestibility, primarily focusing on threonine (Thr) and lysine (Lys), between young and older adults. The protein sources milk, sorghum, and black beans were selected. These protein sources represent animal, cereal, and legume proteins and range in reported true ileal digestibility from >90% for milk [[20], [21], [22]] to <80% for both sorghum and black beans [[22], [23], [24]].

## Methods

The experimental protocol was approved by the Medical Ethical Committee from the Netherlands East and registered in July 2022 at clinicaltrials.gov: NCT05534464. All participants gave written informed consent. A randomized crossover study was performed in the Netherlands for the 3 protein sources, with test days separated by 2 wk, and the order of the protein sources being randomized using Microsoft Excel by the researcher. The dual tracer method was applied, in which 20 g of intrinsically ^2^H-labeled protein source was consumed simultaneously in a meal with 400 mg of a ^13^C-labeled free AA mixture. The plasma-to-meal ratio of the 2 isotopes for 8 indispensable AAs was calculated as the primary measurement of this method [14]. The measurement reflects at least digestion and absorption, but the dual tracer method relies on assumptions that can affect further interpretation. It is assumed that AAs from both the reference and the test protein source have similar absorption and splanchnic metabolism before entering the systemic circulation, thereby the ratio of enrichments neutralizes the splanchnic metabolism, making digestibility the result of the calculation [25]. When isotopes are labeled at labile positions, the calculated ratios include splanchnic metabolism and cannot be interpreted as digestibility exclusively. The splanchnic metabolism includes transamination that ideally should be corrected for per AA and age group. Transamination correction factors could not be determined in this study but were previously reported to range between 1.002 and 1.081 [14]. These correction factors may substantially be higher for older adults as, for example, 2-fold splanchnic metabolism was reported for Leu [10]. Lys and Thr do not undergo transamination and thereby act as the only interpretive anchors of our result. For all other AAs, we do not report the digestibility but report the isotope plasma-to-meal ratio as the primary measurement of this dual tracer method, which would still need correction for age-specific transamination.

### Study population

The study included 10 young adults (20–35 y) and 10 older adults (65–80 y) with a BMI (in kg/m^2^) between 20.0 and 30.0 and with suitable veins for blood sampling. This sample size was based on an estimated SD of 5, a relevant effect size of 3.5% for the difference in AA digestibility between young and older adults, a power of 0.80, and statistical significance of 0.05. By means of a questionnaire, participants were assessed to be healthy and to have regular eating habits. All participants filled out a food frequency questionnaire (FFQ). Participants were excluded if they experienced chronic disease; had a history of medical or surgical events that may affect gastrointestinal function; used medication that interferes with gastrointestinal function; used harmful drugs; drank alcohol excessively (>14 units/wk and >3/d for females and >21 units/wk and >4/d for males); followed a diet; exercised for >5 h/wk; had low hemoglobin (<7.5 mmol/L for females and <8.5 mmol/L for males); donated blood recently (<2 mo prior to study or <4 mo after study termination); had allergy or intolerance to the tested products; or had participated in other research (<2 mo before the study).

### Test meals

Three different types of intrinsically ^2^H-labeled protein sources were produced: milk, sorghum, and black beans. Intrinsically ^2^H-labeled milk was produced by infusing ^2^H_2_O (D214L, Eurisotop) into the rumen of 1 lactating rumen-fistulated Holstein Friesian cow. Subsequently, milk was processed to skimmed milk powder. This applied labeling method appeared to be less appropriate compared with labeling animal protein sources by provision of already labeled AAs via feed or intravenously [25,26]. Intrinsically ^2^H-labeled sorghum (*Sorghum bicolor*, Dusormil HD19 variety) and black beans (*Phaseolus vulgaris*, Coco Noir) were produced by watering the plants with ^2^H_2_O during flowering. Details for the macronutrient and AA composition of all sources are presented in Supplementary Table 1. Details of the labeling experiment are described in the Supplemental Methods and Supplementary Table 2 to 5. Because of a low yield of the first batch of black beans, a second batch was also produced. The reference AA source was purchased: ^13^C-labeled free AA (99%, Eurisotop).

Three different types of test meal were prepared, each containing 20 g of ^2^H-labeled protein from each protein source and 400 mg ^13^C-labeled free AA. To keep the carbohydrate content similar across test meals, complementary products were served along with the milk and the black beans. Skimmed milk powder was dissolved in water and served with a protein-free cookie. Whole-grain sorghum was cooked and mashed. Black beans were soaked overnight, cooked, mashed, and served with a carbohydrate drink (Table 1). More details of the test meal preparation and composition are described in the Supplementary Methods and Supplementary Figure 1.

**TABLE 1:** Composition of the test meals: dry weight of the 3 protein sources with the amount of water added to dissolve (milk) or to allow cooking (sorghum and beans), and prepared weight of the complementary food sources

Protein source (g/meal)			Complementary food (g/meal)		Composition whole test meal (g/meal)		
Source	Quantity	Water added	Source	Quantity	Protein	Fat	CHO
Skimmed milk powder	57.3	573	Protein-free cookie	190.1	20.2	34.1	155.6
Sorghum	214.6	1717	None		20.0	9.2	155.6
Beans (1st batch)	98.5	985	CHO drink	192.3	20.2	1.7	155.6
Beans (2nd batch)	93.5	935	CHO drink	201.0	20.2	1.8	155.6

Macronutrient composition of the complete test meal: consisting of a protein source with the complementary product.

Abbreviation: CHO, carbohydrate.

### Experimental protocol

**FIGURE 1:**
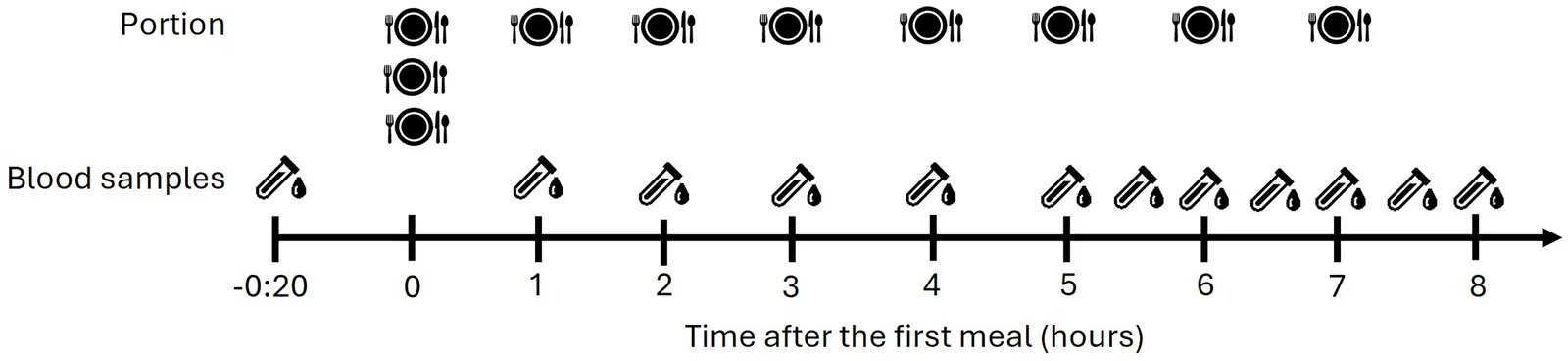
Schematic overview of the experimental protocol on the test day. Hourly portions provided 20 g of protein for each source in total. Blood samples were taken at baseline and frequently after consumption of the first portion.

The experimental protocol for each of the 3 test days is shown in Figure 1. The day before each test day, participants were asked to refrain from foods high in ^13^C (e.g., maize, millet, sugar cane, and pineapple); to refrain from intense physical activity; to consume the same evening meal before each test day; and to remain fasted from 22:00 onward. On each test day, participants arrived at the research facility of Wageningen University, and a venous catheter was inserted in an antecubital vein for blood sampling. Test meals were provided to the participants in 10 portions. The first serving corresponded to 3 portions as a priming dose, followed by equal hourly portions for 7 h to reach an isotopic steady state in the final hours. One extra portion was prepared for meal enrichment analysis. Blood was sampled at baseline and frequently for 8 h after the first serving of the test meals. Samples were taken at hour 1.0, 2.0, 3.0, 4.0, 5.0, 5.5, 6.0, 6.5, 7.0, 7.5, and 8.0. For isotope enrichment analysis, whole blood was transferred into lithium heparin tubes and centrifuged at 2000 × *g*, at 4°C for 10 min to separate plasma. For gut health marker analysis, whole blood sampled at baseline was transferred into EDTA tubes and centrifuged at 1000 × *g* at 4°C for 10 min to separate plasma. Plasma samples were stored at –80°C until analysis.

### Sample analysis

^2^H- and ^13^C-enrichments of 8 indispensable AAs (histidine, isoleucine, Leu, Lys, Phe, methionine, Thr, and valine) were determined in the test meals and in the plasma samples for each participant. Enrichment was expressed in atom percent excess with enrichment in meals and plasma corrected for enrichment in the baseline plasma sample, as not an isotopically labeled reference. ^2^H-enrichment analysis was performed by gas chromatography-pyrolysis-isotope ratio mass spectrometry and ^13^C-enrichment analysis was performed by gas chromatography-combustion-isotope ratio mass spectrometry. Details of the analysis can be found in the Supplemental Methods.

Plasma from the baseline sample was analyzed for gut health markers, intestinal fatty acid binding protein (I-FABP), and zonulin. Levels of I-FABP were determined using an ELISA according to the manufacturer’s instructions (HK406, Hycult Biotech) with 2 times dilution of plasma. Plasma levels of zonulin were determined using ELISA, according to the manufacturer’s instructions (HUES03536, AssayGenie), with 10 times dilution of plasma. The concentrations were obtained via a 4-parameter logistic curve using the ELISA calculation sheet on the company’s website (HycultBiotech). The samples were analyzed in duplicate.

### Data analysis

Protocol deviations and adverse events were noted during the test days. Where major protocol deviations occurred, data from a complete test day were excluded from further analysis, whereas for minor protocol deviations, the data were assessed for their impact on the outcome measures occurred. Excluded data from protocol deviations were not included in further analyses. Participants with a few missing blood samples were still included in the analysis. Steady state for plasma enrichment was defined as the average over timepoints between 5.5 and 8.0 h (6 samples). To check steady states systematically, the slope over time as a percentage of the mean from the selected samples was calculated. When this value was >15%, timepoints were removed from the 5.5 h timepoint to later timepoints until the value became <15% with ≥3 samples left. If no slope with a value <15% was reached, the graphs were visually inspected by 2 researchers to determine the steady state and decide whether timepoints should be excluded other than from 5.5 h onward. These average plasma enrichments at steady state combined with meal enrichments of ^2^H and ^13^C were used to calculate the isotope plasma-to-meal ratio as a measure for digestibility for each of the 8 indispensable AAs per participant:

Hplasma2Cplasma13/Hmeal2Cmeal13=isotopeplasma−to−mealratio

As an additional analysis, the AUC for plasma enrichment was determined over the full postprandial period (11 time points) and used for isotope plasma-to-meal ratio calculations instead of the steady state values.

### Statistical analysis

Data were processed using Microsoft Excel and RStudio (2024.04.2). Descriptive statistics are presented as mean and SD. Because we were not able to determine transamination factors for the different age groups in our study, we only considered Thr and Lys as the 2 nontransaminating AA. To test the difference in isotope plasma-to-meal ratio between age groups and protein sources for Lys and Thr, a linear mixed model was applied where age group, protein source, and AA (Lys and Thr) were added as fixed factors and participants as a random factor. Interactions included in the model as additional fixed factors considered Age∗Source and Source∗AA. No other covariates were added to the model. The model was checked for normality of residuals and homoscedasticity using QQ plots and residual plots. Pairwise comparisons and Bonferroni’s correction for multiple comparisons were performed as post hoc analysis. For all the comparisons, *P <* 0.05 was considered significant. Results from the linear mixed model analysis are presented as estimated marginal means (EMM) along with their SE from the model.

## Results

**FIGURE 2:**
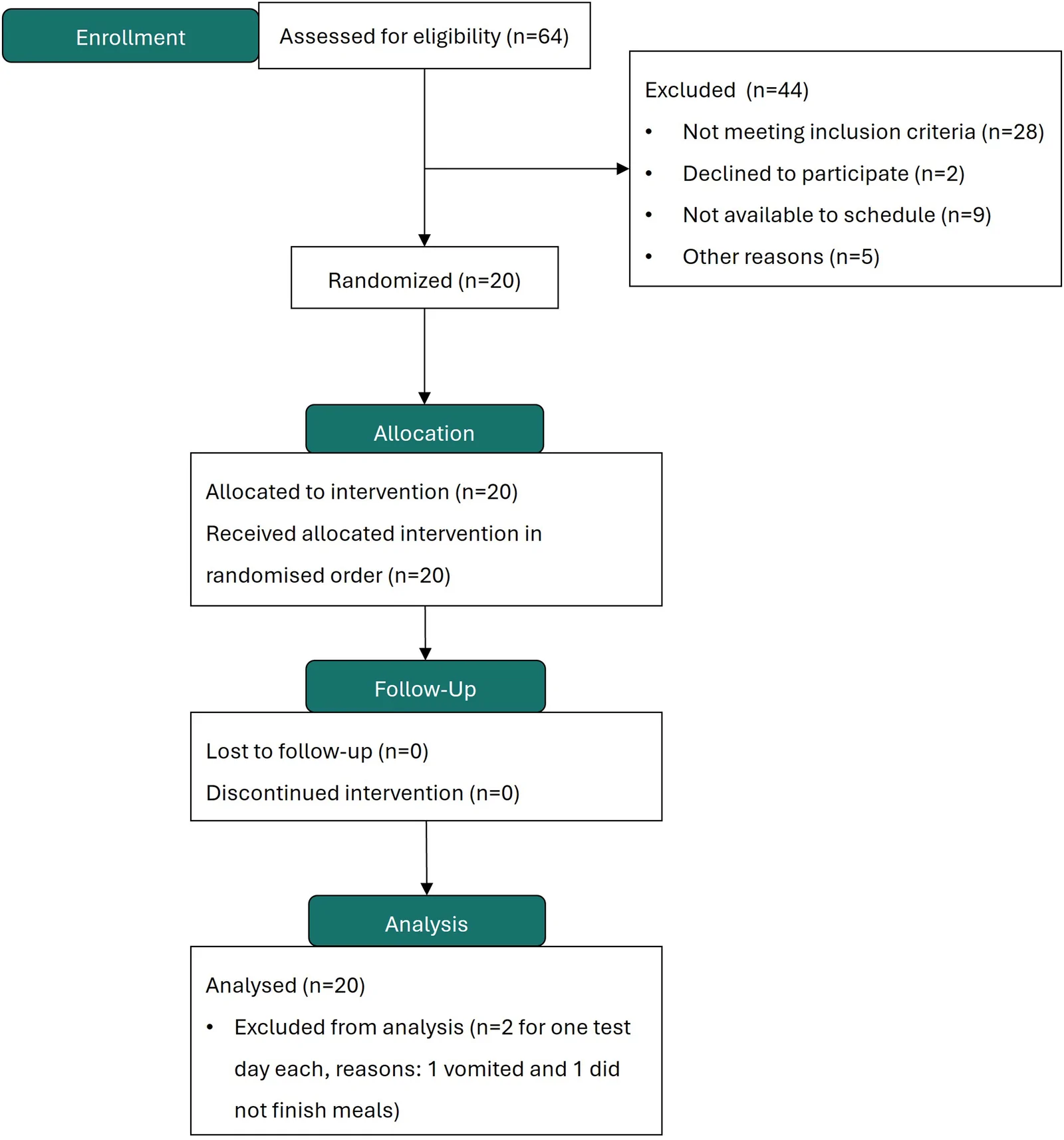
CONSORT flow diagram of participant enrollment, follow-up, and analysis.

Participant recruitment and study execution were from September 2022 to April 2023. The flow chart of participant inclusion is presented in Figure 2. The study population baseline characteristics are presented in Table 2, with habitual energy and protein intake derived from the FFQ. By definition, older adults had a higher age, but also a higher BMI. Overall, absolute habitual protein intake was similar, whereas relative protein intake, expressed as a percentage of energy, was higher for the older adults. Older adults also consumed less protein from plant sources and tended to consume more protein from animal sources compared with younger adults. Fasting glucose and gut health markers were comparable.

**TABLE 2:** Baseline characteristics study population (mean ± SD)

	Young	Older
n	10	10
Sex ratio (M/F)	4/6	5/5
Age (y)	21.8 ± 1.7	72.8 ± 3.8
Height (cm)	178.2 ± 9.9	174.6 ± 10.4
Weight (kg)	70.5 ± 6.9	76.8 ± 11.7
BMI (kg/m^2^)	22.2 ± 2.1	25.1 ± 2.5
Hb (mmol/L)	8.5 ± 0.7	8.6 ± 0.7
Fasting glucose (mmol/L)	4.8 ± 0.3	5.0 ± 0.4
Habitual energy intake (kcal/d)	2311 ± 478.0	2116 ± 662.7
Habitual protein intake (g/d)	76.3 ± 20.0	79.1 ± 22.5
Habitual protein intake (en%)	13.1 ± 1.1	15.1 ± 1.5
Animal protein intake (g/d)	33.3 ± 16.1	47.0 ± 15.4
Plant protein intake (g/d)	43.0 ± 11.6	32.0 ± 10.9
Zonulin (ng/mL)	154 ± 206	146 ± 187
I-FABP (pg/mL)	785 ± 258	809 ± 284

Abbreviations: F, females; Hb, hemoglobin; I-FABP, intestinal fatty acid binding protein; M, males.

**FIGURE 3:**
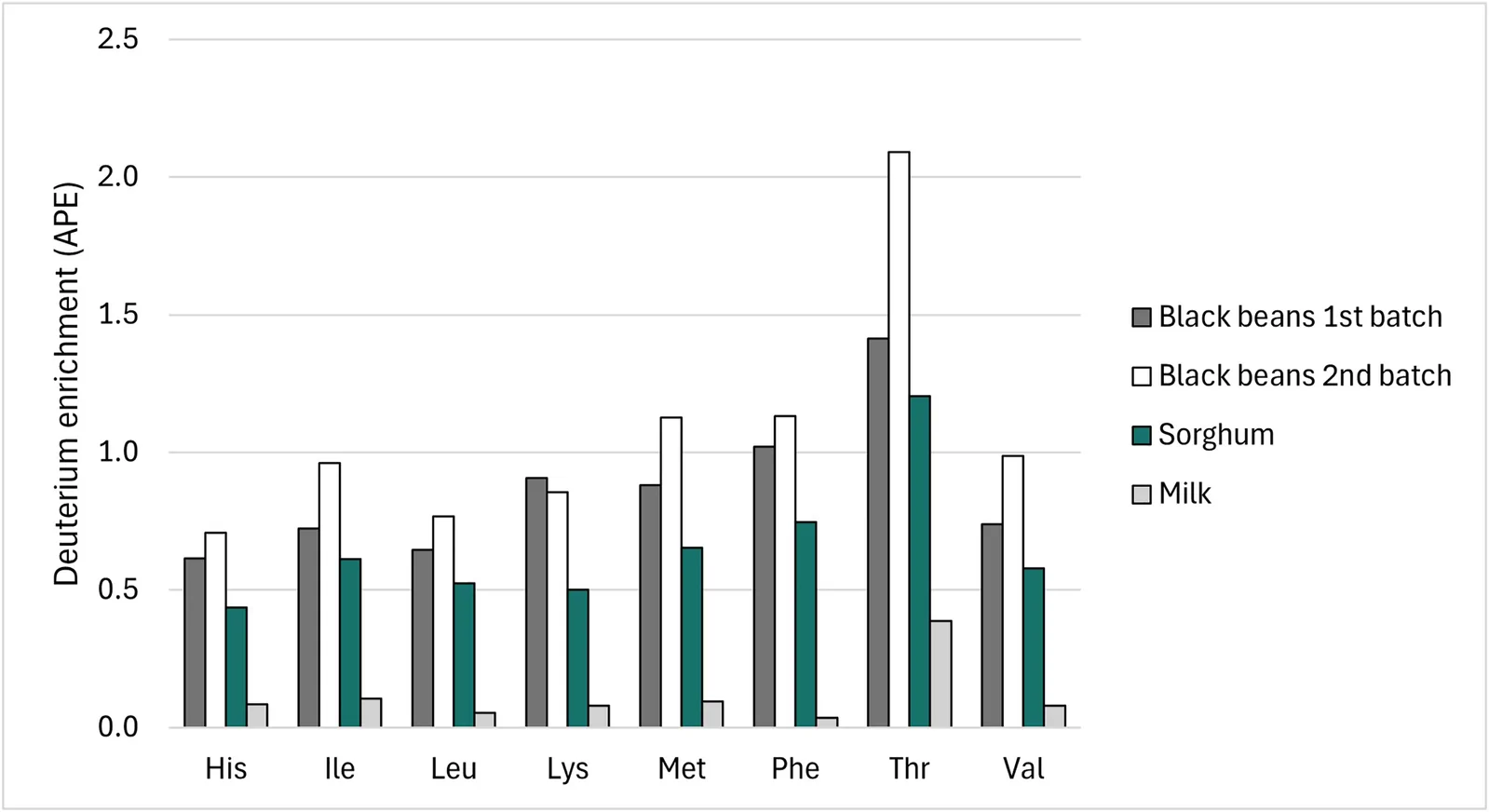
^2^H-enrichment of indispensable amino acids in APE for milk, sorghum, and black beans after intrinsic labeling with deuterated water. APE, atom percent excess; His, histidine; Ile, isoleucine; Leu, leucine; Lys, lysine; Met, methionine; Phe, phenylalanine; Thr, threonine; Val, valine.

All 3 protein sources were sufficiently enriched after intrinsic labeling with deuterated water. The ^2^H-labeling was, however, not homogeneous across the AAs for the 3 protein sources: Thr was the most highly enriched AA (Figure 3). Furthermore, the plant protein sources showed higher enrichments than milk, and the second batch of black beans showed higher enrichments than the first batch of black beans. The ^2^H- and ^13^C-enrichments in the test meals served to participants are presented in Table 3. The difference in ^2^H-enrichment between the protein sources and the meals for some AAs may be due to the loss of labeled positions during cooking and hydrolysis.

**TABLE 3:** Determined meal ^2^H- and ^13^C- AA enrichments (APE) in test meals (mean ± SD) for milk (*n* = 19), sorghum (*n* = 19), and black beans (*n* = 20: *n* = 10 per batch)

	^2^*H*			^13^*C*		
	Milk	Sorghum	Black beans	Milk	Sorghum	Black beans
Lys	0.069 ± 0.002	0.371 ± 0.019	0.753 ± 0.113	0.208 ± 0.016	0.632 ± 0.076	0.207 ± 0.044
Thr	0.087 ± 0.006	0.628 ± 0.040	0.946 ± 0.128	0.771 ± 0.047	0.849 ± 0.089	0.729 ± 0.139
His	0.049 ± 0.002	0.267 ± 0.018	0.485 ± 0.091	0.115 ± 0.038	0.135 ± 0.054	0.143 ± 0.038
Ile	0.102 ± 0.003	0.541 ± 0.030	0.858 ± 0.191	1.236 ± 0.084	1.169 ± 0.092	1.209 ± 0.251
Leu	0.056 ± 0.003	0.504 ± 0.031	0.773 ± 0.158	0.963 ± 0.065	0.544 ± 0.044	1.016 ± 0.217
Met	0.101 ± 0.005	0.547 ± 0.045	0.923 ± 0.118	0.378 ± 0.029	0.451 ± 0.041	0.526 ± 0.096
Phe	0.036 ± 0.012	0.578 ± 0.036	0.986 ± 0.167	1.528 ± 0.097	1.164 ± 0.107	1.130 ± 0.233
Val	0.073 ± 0.002	0.535 ± 0.030	0.884 ± 0.175	0.983 ± 0.058	0.984 ± 0.080	1.034 ± 0.198

Abbreviations: AA, amino acid; APE, atom percent excess; His, histidine; Ile, isoleucine; Leu, leucine; Lys, lysine; Met, methionine; Phe, phenylalanine; Thr, threonine; Val, valine.

Major protocol deviations related to 1 young adult participant who did not finish the sorghum meals and 1 older adult participant who felt sick and vomited. Both deviations resulted in a disturbed plasma enrichment steady state, and these 2 test days were removed from the analysis. For Thr in milk, 2 observations had an outcome >1.4 and were removed from statistical analysis. Minor protocol deviations (e.g., bladder infection with antibiotic use) were retained in the dataset, and these data did not impact the conclusions significantly. Our method for assessing a steady state between 5.5 and 8.0 h requested adjustment in 14% of the cases. In these cases, the steady-state value was based on fewer data points. The AA ^2^H- and ^13^C-enrichments over time in plasma at steady state for each protein source and age group are presented in Supplementary Figures 2 and 3. Fluctuations observed represent the hourly feeding and half-hourly sampling, but this did not impact the defined steady state. The exclusion of timepoints did not affect the main conclusions on the effects of age on Lys and Thr digestibility; results based on the full dataset are in Supplementary Figure 4 and Supplementary Table 6.

**FIGURE 4:**
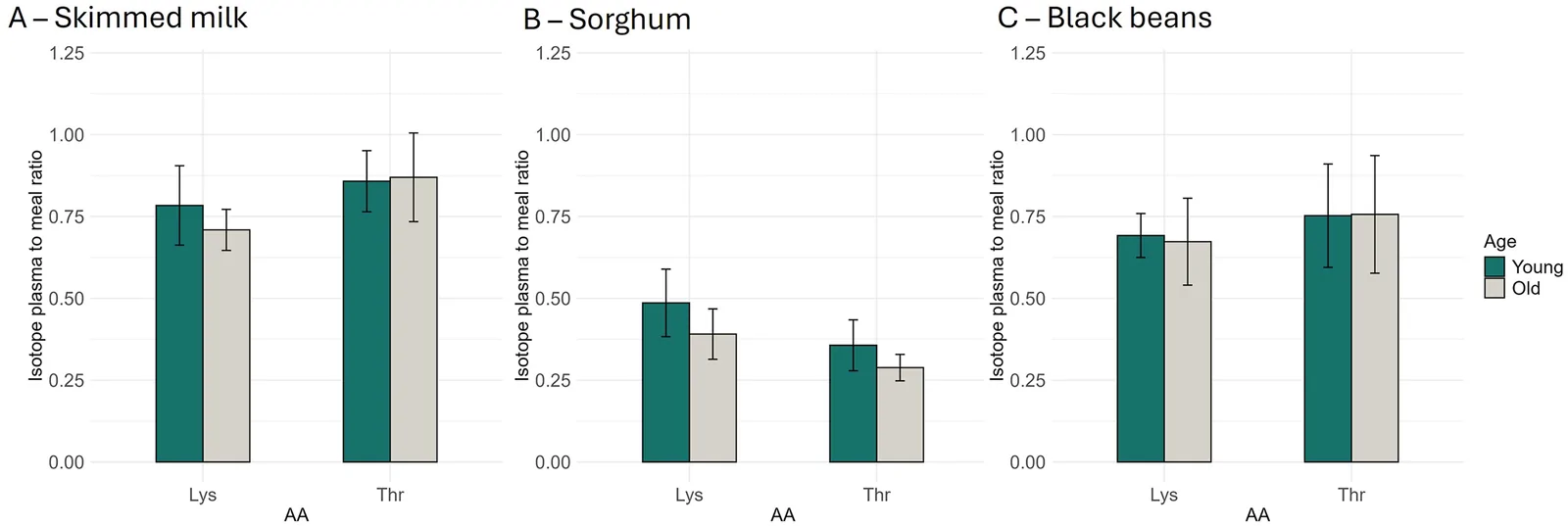
Isotope plasma-to-meal ratio (mean with SD), for Lys and Thr separated for young and older adults for milk (A; young *n* = 10; old *n* = 9, for Thr young *n* = 8), sorghum (B; young *n* = 9; old *n* = 10) and black beans (C; young and old *n* = 10). AA, amino acid; Lys, lysine; Thr, threonine.

Isotope plasma-to-meal ratio derived at steady state for Lys and Thr in the 3 protein sources and the 2 age groups is depicted in Figure 4. Because these 2 AAs do not undergo transamination, their isotope plasma-to-meal ratios are a direct representation of digestibility and can be compared between the 2 age groups. We were not able to apply age-specific transamination correction factors; the isotope plasma-to-meal ratio of all other indispensable AAs is shown in Supplementary Figure 5.

The linear mixed model did not show an overall difference between young and older adults in steady state plasma-to-meal ratio averaged for protein sources and Lys and Thr (main effect age: –0.045, *P* = 0.135). Table 4 displays EMM from the model. Post hoc analysis showed that the ratio averaged over Lys and Thr was 19% lower in older adults compared with young adults for sorghum (*P* = 0.041, Δ = –0.08, confidence interval (CI) = –0.16, –0.004), whereas it was not significantly different for milk (*P* = 0.277, Δ = –0.04, CI = –0.13, 0.04) and black beans (*P* = 0.849, Δ = –0.007, CI = –0.09, 0.07). Although not standard for the dual tracer approach, an additional plasma-to-meal ratio was calculated using the AUC (Supplementary Figure 6). The AUC plasma-to-meal ratio as a measure of Lys and Thr digestibility also showed no difference between age groups (*P* = 0.687). In contrast to the results derived from steady state, post hoc analysis did not show any significant differences between age groups per protein source (Supplementary Table 7).

**TABLE 4:** Estimated marginal means for steady-state isotope plasma-to-meal ratio of lysine and threonine in milk, sorghum, and black beans for young and older adults

	Milk		Sorghum		Black beans	
	Young	Old	Young	Old	Young	Old
Lys	0.77	0.72	0.48	0.40	0.69	0.68
Thr	0.88	0.84	0.37	0.28	0.76	0.75
SE	0.03	0.03	0.03	0.03	0.03	0.03
P	0.278		0.041		0.849	

Abbreviations: Lys, lysine; Thr, threonine.

## Discussion

This study aimed to investigate whether AA digestibility is decreased on aging. Using the dual tracer approach, we showed that the isotope plasma-to-meal ratio for Thr and Lys was significantly lower in older adults compared with young adults for sorghum (19%), but not for milk and black beans.

The dual tracer method is an innovative method positioned to indirectly determine AA digestibility of food proteins in humans with the use of stable isotopes. Several studies using the dual tracer method have reported digestibility values that are similar to those reported in the literature as determined with other methods [27,28], but for accurate digestibility coefficients, the method has not been validated formally [15]. Recent studies also showed that the dual tracer method resulted in ∼10% lower digestibility coefficients compared with the oro-ileal balance method for Lys in sunflower protein [17], casein and pea protein [16], and milk protein [18], although these studies have some limitations in the applied feeding pattern and isotope selection that makes determination of isotopic steady state and proper transamination correction difficult. The calculated isotope plasma-to-meal ratios incorporate processes of digestion and absorption, and splanchnic metabolism. Therefore, these values provide a measure for the digestive process and any physiological changes with aging. The rate and extent of transamination may be affected by aging in humans, because transamination processes take place during splanchnic metabolism, which is increased with aging [10,11]. Because our results are not corrected for transamination, which is a limitation of this study, we focused on the nontransaminating AAs Lys and Thr in our analysis and interpretation.

The isotope plasma-to-meal ratio for Lys in black beans of 0.69 ± 0.04 in younger and 0.68 ± 0.04 in older adults is lower compared with true ileal Lys digestibility coefficients 0.82 for total Lys and 0.86 for reactive Lys in cannulated pigs [22]. Thr (0.76 ± 0.03 in younger and 0.75 ± 0.03 in older adults) showed more similar values to true ileal Thr digestibility of 0.71 in cannulated pigs [22].

The isotope plasma-to-meal ratio for skimmed milk was similar in younger compared with older adults for Thr, 0.88 ± 0.03 compared with 0.84 ± 0.03, and for Lys, 0.77 ± 0.03 compared with 0.72 ± 0.03. The outcome values are, particularly for Lys, lower compared with previous digestibility reports for a similar protein source. In humans, a digestibility of 89.9% ± 1.2% for Thr and 93.0% ± 2.1% for Lys was reported for goat milk using the dual tracer method [26] and a true ileal protein digestibility of 95.5% ± 0.4% for ^15^N-labeled milk with nasoileal intubation [21]. In ileal cannulated pigs, the Thr ileal digestibility ranged between 82% and 96% for milk protein concentrate, skimmed milk powder [20], dry milk [29], pasteurized milk, and high temperature treated milk [30]. Lys ileal digestibility in cannulated pigs ranged between 95% and 98% for milk protein concentrate, skimmed milk powder [20], dry milk [29], ^15^N-labeled milk protein concentrate [18], pasteurized milk and high temperature treated milk [30] measured with oroileal balance method, whereas it was 88.9% for ^15^N-labeled milk protein concentrate with the dual tracer method [18]. The observed lower and more variable isotope plasma-to-meal ratios for all other AAs may arise from our applied intrinsic labeling protocol for milk. We infused ^2^H_2_O directly in the cow’s rumen, which will primarily result in ^2^H-labeling of indispensable AAs through transamination exchanges of AA in this heterotrophic animal [25]. Therefore, a larger part of the ^2^H-label in the AAs of milk is labile and may disappear at a higher rate in postprandial transamination processes. With this knowledge, it is expected that higher transamination correction factors are needed for milk compared with sorghum and black beans. The latter were labeled through de novo AA synthesis in the autotrophic plant protein sources [25]. Transamination correction factors for deuterium have been determined and applied for autotrophically labeled protein sources [14]. In the current study, transamination factors could not be determined, and results were not corrected for transamination [26].

Data for sorghum indicated poor digestibility with an isotope plasma-to-meal ratio of 0.48 ± 0.03 in younger and 0.40 ± 0.03 in older adults for Lys and 0.37 ± 0.03 in younger and 0.28 ± 0.03 in older adults for Thr. Variety and processing of sorghum grains can have major effects on their digestibility. Fully processed sorghum flour had a higher true ileal digestibility of 0.88 for total Lys, 0.91 for reactive Lys, and 0.82 for Thr in cannulated pigs [22], whereas sorghum flour had a high variable overall AA digestibility in human ileostomates ranging from 0.45 to 0.88 [31]. Unprocessed whole-grain sorghum showed a wide range for in vitro protein digestibility from 9% to 81% using the INFOGEST method [32]. The tannin-free genotype HD19 variety, as used in the current study, showed the highest in vitro digestibility value [32]. Cooking this sorghum led to a reduction in protein digestibility of 33%, whereas dehulling grains can diminish this effect toward a reduction of 12% [32]. Cooking sorghum grains was shown to reduce in vitro protein digestibility to ∼30% [33,34]. Starch and protein are closely associated within the sorghum grain, where cooking sorghum forms resistant starch, which can make the protein less accessible for enzymatic hydrolysis [33,35,36]. Low values for cooked, whole-grain sorghum align with our results for Lys and Thr in sorghum. The difference in measuring methods could explain the lower values as well.

The dual tracer method relies on assumptions that can affect the interpretation of the outcome measure. To meet the assumption of similar kinetics and splanchnic metabolism from test and reference protein [25], the most suitable reference for this method is debated [15]. We selected a mixture of free AA over ^13^C-spirulina to prevent a potentially reduced digestive capacity in older adults, to equally affect the reference protein as our test protein. Slight differences in splanchnic metabolism between the free AAs of the reference and dipeptides and tripeptides of the test protein have been suggested to potentially result in deviations from real digestibility [25]. Concerning the assumption of steady state [15], our results of plasma isotope enrichment over time reflected the hourly feeding and half-hourly sampling pattern, which shows similar fluctuations to previous studies [27,28,37]. However, feeding portions more frequently, for example, every 20 min, would be recommended for future studies to establish steady states with less fluctuations and to prevent potential differences in metabolism of the test and reference AA. Alternatively, the AUC plasma-to-meal ratio showed lower values and did not show any difference between young and older adults. This method takes the whole postprandial period into account, which is sensitive to differences in gastric emptying, which changes with aging [8]. Because a plateau feeding protocol is applied, the steady state calculation is preferred. When steady state is not achieved, the AUC can be an alternative option for calculation, and it was previously shown to provide similar results [16]. A limitation of our analysis was that the sample size calculation was not powered for all the interactions tested.

This study showed a lower isotope-plasma-to-meal ratio of Lys and Thr, as a measure of the digestive process, in older compared with young adults for sorghum but not for black beans or milk. We are the first aiming to compare AA digestibility between young and older human individuals, showing a 19% difference for one protein source. In rats, a 5% to 17% difference in fecal protein digestibility between young and older rats was found, likely depending on the ANF content of the protein sources [12]. It could be hypothesized that older adults are more sensitive than younger adults to ANF content in combination with processing that decrease the digestibility potential of protein sources, for example, because of lower enzyme concentrations. Sorghum contains several ANFs such as tannins and phytate [38,39] and cooking sorghum leads to decreased protein accessibility for enzymes. Both factors together could explain our observed difference between young and older adults. More detailed information is needed about the ANF content and effect of processing in our protein sources before drawing further conclusions [23].

To conclude, these differences in isotope plasma-to-meal ratio suggest that Lys and Thr digestion is lower for older adults for sorghum, but not for black beans and milk. Reduced digestion of this protein source increases the risk for protein and AA malnutrition in older adults. Given our results suggest there may be differences between protein sources, one possible avenue for future recommendations could be to consider exploring the development of an age-group-adjusted protein quality score. However, more work is needed to extend our results by investigating the effect of aging on AA digestibility in multiple protein sources, in which the ANF content and processing of the protein source requires attention. Furthermore, the use of medication and disease state could greatly impact protein digestibility, which needs investigation as our results were representative of a healthy older adult study population. Overall, the findings contribute to provide tailored protein recommendations for older adults to meet their AA requirements.

## Author contributions

The authors’ responsibilities were as follows – NvdW, MM, TH: designed research; RT, FH: conducted research and analyzed data; DvH: performed laboratory analysis; FH: wrote the article; and all authors: read, improved, and approved the final manuscript.

## Data availability

Data described in the manuscript, code book, and analytical code will be made available on request pending.

## Declaration of generative AI and AI-assisted technologies in the writing process

During the preparation of this work, the authors used ChatGPT to optimize statistical R code and provide textual editing suggestions. After using this tool, the authors reviewed and edited the content as needed and take full responsibility for the content of the publication.

## Funding

This work is part of the public–private partnership project “Quantifying differences in bioavailability of different dietary proteins in older adults” cofinanced by the Top Consortium for Knowledge and Innovation Agri & Food by the Dutch Ministry of Economic Affairs under contract number LWV20155 and a consortium of partners (Nestlé, Dairy Management Inc., FrieslandCampina, Arla Foods amba).

## Conflicts of interest

TH is employed by the company FrieslandCampina. The research was conducted without any relationships with the company that could be construed as potential conflicts of interest. No potential conflict of interest was reported by the other authors.
