# Supplementary material for: Differences in amino acid digestibility between young and older adults: a randomized crossover study using the dual tracer method
Source: Am J Clin Nutr. 2026 May 19;124(1):101361. doi: 10.1016/j.ajcnut.2026.101361 (PMC13494179; doi:10.1016/j.ajcnut.2026.101361)
Supplement: Supplementary file 1 [file mmc1.docx]

## SUPPLEMENTARY METHODS

### Food composition

Macronutrient composition of test protein sources (Supplementary Table 1) was determined for protein with Dumas, for fat after acid hydrolysis and petroleum ether extraction, and for carbohydrates by estimation. AA composition (Supplementary Table 1) of test protein sources was determined according to ISO13904 and ISO13903.

### Intrinsic ^2^H-labelling of protein sources

Deuterated water (Eurisotop, France, D214L) was infused directly into the rumen of one rumen-fistulated Holstein Friesian cow of 6 years old in her fourth lactation period. The labelling protocol can be found in Supplementary Table 2. The protocol started on July 20 2022 and the milk collected on the two days after the labelling protocol ended (July 23 and 24 2022) was used for the experiment. This milk was defatted (50 ⁰C), pasteurized (72 ⁰C) and spray-dried into skimmed milk powder and analyzed for microbial quality and other contaminants at FrieslandCampina (Wageningen, the Netherlands). The canned milk powder was stored at -20 ⁰C until use.

Sorghum (*Sorghum bicolor*, Dusormil HD19 variety, kindly provided by Walter de Milliano) seeds were sown in greenhouses of Unifarm (Wageningen University and Research). 500 germinated plants were transferred to 250 pots. The mean daily measured temperature was 25.5 ± 1.4 °C during the day and 20.6 ± 0.6 °C during the night with a range of daily temperature of 21.4-30.3 °C during the day and 17.3-21.9 °C during the night. The mean daily humidity was 53.4 ± 11.2% during the day and 65.9 ± 11.8% during the night. 12 L pots were filled with Unifarm standard potting soil (n°4). Watering and daily management were left to Unifarm personnel. Some biological control took place during the experiment, both curative and preventive. When the sorghum heads emerged, the plants received deuterated water (Eurisotop, France, D214L). The labelling protocol can be found in Supplementary Table 3. The plants were watered on the days between the intake days of deuterated water. Sorghum was harvested from the plants and heads were dried for a week. The heads were threshed to collect sorghum grain, and the mean yield was 22.0 gram/head. Sorghum samples were analyzed for food safety at SGS (Spijkenisse, the Netherlands). Sowing, labelling and harvest took place between March and July 2022. Sorghum was stored dry and at room temperature.

Black beans (*Phaseolus vulgaris*, Coco Noir) seeds (Vreeken’s Zaden, The Netherlands) were sown in greenhouses of Unifarm (Wageningen University and Research). The 168 germinated seedlings were transferred to 84 pots. Climate settings were 24 °C during the day and 20 °C at night with a natural day length. The mean daily measured temperature was 27.0 ± 2.5 °C during the day and 21.1 ± 0.9 °C during the night with a range of daily temperature of 21.9-36.3 °C during the day and 19.1-24.9 °C during the night. The mean daily humidity was 53.5 ± 15.7% during the day and 69.9 ± 11.7% during the night. 12 L pots were filled with Unifarm standard potting soil (n°4). Watering and daily management were left to Unifarm personnel. Some biological control took place during the experiment, both curative and preventive. When flowers appeared, the bean plants received deuterated water (Eurisotop, France, D214L). The labelling protocol can be found in Supplementary Table 4. Sowing, labelling and harvest took place between April and July 2022 The harvest yielded only enough beans for the first ten participants. Therefore, a second batch of beans was sown and the 212 strongest plants were transferred in 106 pots. These plants underwent the labelling protocol depicted in Supplementary Table 5. The harvest of this new batch yielded enough for the last ten participants. Sowing, labelling and harvest took place between June and August 2022. Bean samples were analyzed for food safety by SGS (Spijkenisse, the Netherlands). Beans were stored dry and at room temperature.

### Test meal preparation

Some preparation for the protein sources was performed the day before the test day. Milk powder was taken from the freezer and protein free cookies were baked. Eleven protein free cookies contained 95.5 g purified corn flour, 46.0 g margarine, 53.4 g sucrose, 11.5 g cellulose, 1.6 g baking powder and 1.2 g ground ginger and were baked at 190 ⁰C for 50 minutes. Per 100 g the cookies contained 0.1 g protein, 17.7 g fat and 66.0 g carbohydrates according to the food composition table. Sorghum was cooked with water in 1:8 ratio for 80 minutes to ~600 grams, it was mashed with an electric mixer (Dynamic MD95) and stored in the fridge. Black beans were soaked overnight with water in 1:2 ratio, cooked in water in 1:8 ratio for 60 minutes to ~300 g, mashed with an electric mixer (Dynamic MD95) into a puree (Supplementary Figure 1) and stored in the fridge.

On the test day the milk powder was diluted with water in 1:10 ratio. Lemonade syrup (Karvan Cevitam) was mixed with water in 1:7 ratio to ~1800 mL carbohydrate drink. According to the label the syrup contained 0.1g protein, 0.0g fat and 49.4g carbohydrates per 100g. Portions of sorghum and beans were heated in the microwave (Panasonic NE1880) for 30 seconds at 1800 W. ^13^C-labelled free amino acids were weighed per portion on an analytical scale (Sartorius, Practum 224) and mixed with each portion separately after heating. Complementary products were served next to the protein source. In total, eleven portions were prepared of which ten were provided to the participants and contained 20 grams of protein in total and the eleventh portion was prepared for analysis of meal enrichment.

### Laboratory analysis ^13^C-enrichment of amino acids

In each blood sample, ^13^C-enrichment in plasma AA was measured according to Kampman-van de Hoek (1). AA were extracted from plasma by solid phase extraction and dissolved in an ammonia solution. In detail, 20 μl hydrogen chloride (1M), 200 μl Dowex ion exchange resin solution (AG 50W-X8 Hþ form, 200–400 mesh; Dow Chemical Company) and 20 μl norleucine (1 mmol/l) were added to 180 μl plasma. After washing, neutralizing, the Dowex twice with 1 mL water, the amino acids were eluted with 0.7 mL ammonium hydroxide (6M). The supernatant was evaporated with a centrifugal concentrator (Speedvac SSP 131 DDA; Thermo Scientific) under vacuum at 45 ⁰C for 2 hours. After drying, the sample was resolved with 200 μl water. Derivatization was performed by adding 140 μl ethanol–pyridine (4:1, v/v) and 20 μl ethyl-chloroformate (ECF; Merck Schuchardt OHG) to the sample. Derivates were extracted by adding 400 μl hexane–dichloromethane–ECF (50:50:1 by volume). After centrifuging, 200 μl supernatant was transferred in a vial. Additionally, 400 μl hexane–dichloromethane–ECF (50:50:1 by volume) was added, centrifuged and 400 μl supernatant was transferred into the same vial. The supernatant in the vial, in total 600 μl, was dried under N_2_ gas at room temperature. After evaporation, 20 μl pyridine and 50 μl acetic acid anhydride was added to the vial, mixed and closed with a cap. The sample was placed in an alumina block for 1 hour at 60 °C. When the sample was cooled down, the cap was removed, and the solvent was evaporated under nitrogen flow for about 5-20 minutes to dryness. 50 μl of ethyl acetate was added, after mixing transferred to insert and put in a new vial and closed with a cap. The ^13^C-enrichment was determined with GC-C-IRMS. The sample (1 μl) was injected, with PTV injection (Program Temperature Vapour) on the GC. AA were separated by GC (Trace Ultra GC, Thermo Scientific, Milan, Italy, column no. CP8982, VF-17 ms 30 m X 0.25 mm, film 0.25 mm; Agilent Technologies) and the ratio between ^13^C and ^12^C in the individual AA were determined by the connected IRMS (Delta V Advantage, Thermo Scientific, Bremen, Germany) and combustion (Combustion III, Thermo Scientific). The ratio is expressed as delta units which is the ‰ difference with an international reference value. A large batch of plasma sample was used as a control sample. The control sample was prepared the same way as the other samples and analyzed in each series. The samples were measured in simplo. The limit of detection is 100mV and the limit of quantification is 300 mV, the maximum measurement is 50 V.

For AA enrichment analysis in meals, the lyophilized meals were hydrolyzed. From the ball-mill ground feedstuff, 5 mg was weighed in a small glass tube and 1 mL 6M hydrochloric acid was added. With a special gas burner, works on methane and oxygen, a capillary was made on the top of the tube. The capillary was connected to a vacuum pump. When the vacuum in the tube reached about 24 mbar the capillary was closed inside a flame from a Bunsen burner. Hydrolysis took place for 23 hours at 110 °C in an oven. After cooling down, the top of the tube including the capillary was removed. The hydrochloric acid was evaporated with a centrifugal concentrator (speedvacSSP 131 DDA; Thermo Scientific) under vacuum at 75 °C for 2 hours with lamp on. The dry sample was resolved with 2 mL 0.1M hydrochloric acid and filtrated by a 0.2 μm syringe filter. From this sample 180 μl was taken and 20 μl norleucine (1 mmol/l) was added (no additional hydrochloric acid was needed), followed by the procedure for the plasma analyses in blood.

### Laboratory analysis of ^2^H-enrichment of amino acids

For deuterium enrichment analysis, AA in plasma were derivatized using an adaptation of a previously developed method (2). In short, 1 mL of plasma and 35 µL 12M HCl (Merck, Germany) were mixed. Dowex solution (Biorad, Ag 50W-X8 H+ (200-400 mesh), Biorad, Lunteren, the Netherlands) (1:1 (w/w) in doubly distilled water (Elga, High Wycombe, United Kingdom), was prewashed with 1M NaOH and 1M HCl, and washed with water until a pH between 5-7 was achieved. 500 µL of a Dowex was added to the plasma, and the samples were shaken and centrifuged for 2 min at 3000 x *g*. The supernatant was discarded, and the pellet was washed twice by addition of 1 mL of doubly distilled water, shaking and centrifugation for 2 min at 3000 x *g*, and discarding of the supernatant.

Then, 1 mL of a 6 M ammonia (Merck, Germany) was added to elute the AA from the Dowex. The samples were shaken and centrifuged for 2 min at 3000 x *g*, and the supernatant was transferred to a clean vial. The elution step was repeated with 0.5 mL of 6M ammonia, and the supernatant of the second elution step was combined with supernatant of the first elution step. The samples were evaporated to dryness in a speedvac (Labogene ScanSpeed 40, Allerød, Denmark). Then, they were redissolved in 400 µL of doubly distilled water. 280 µL of an ethanol/pyridine (4:1, v/v; Merck, Germany) mixture was added, followed by 40 µL ethyl-chloroformate (Merck, Germany). The mixture was incubated for 5 min at room temperature. This was followed by extraction with 800 µL of a dichloromethane/hexane/ethyl-chloroformate (50:50:1, v/v/v; Merck, Germany) mixture. After shaking and centrifugation for 1 min at 2000 x *g*, 600 µL of the upper layer was transferred in a clean sample vial. The extraction was repeated with 800 µL of the dichloromethane/hexane/ethyl-chloroformate (50:50:1, v/v/v) mixture, and 800 µL of the upper layer was combined with the upper layer from the first extraction step. The solvents were evaporated under a gentle stream of nitrogen at room temperature. The derivates were dissolved in 30 µL of ethyl acetate (Merck, Germany) and transferred to a vial insert. The extracts were then analyzed by GC-p-IRMS (Delta-V isotope ratio mass spectrometer coupled online with a Trace 1310 gas chromatograph and GC Isolink II (Thermo Fisher, Germany)) for deuterium enrichment of valine, leucine, isoleucine, methionine, phenylalanine, lysine, histidine and tryptophan. Then, after initial analysis, the samples were dried under a gentle stream of nitrogen, and 20 µL pyridine (Merck, Germany) and 50 µL of acetic anhydride (Merck, Germany) were added. This was incubated for 1 hour at 60°C. After cooling to room temperature, the solvents were evaporated under nitrogen, and the derivates dissolved in 30 µL of ethyl acetate. The samples were analyzed to determine the threonine enrichments.

Calibration curves were prepared for correction of the analysis results of the samples using: Amino Acid Mix Solution (Sigma, Schnelldorf, Germany), d_3_-histidine (Campro Scientific; Veenendaal, the Netherlands), d_3-_leucine, d_10_-isoleucine, d_4-_lysine, d_3-_methionine, d_5_-phenylalanine, d_5_-^15^N-threonine, d_5_-tryptophan and d_8_-valine (Cambridge Isotope Laboratories Inc., Tewksbury, United States of America). Standards were diluted with 0.1M HCl. Enrichment was determined using a Delta-V isotope ratio mass spectrometer coupled online with a Trace 1310 gas chromatograph and GC Isolink II (Thermo Fisher, Bremen, Germany). Aliquots were introduced into the GC system by a Triplus autosampler. The GC was fitted with a VF17ms column (30 m x 0.25 mm x 0.50 μm, Agilent Technologies, Amstelveen, the Netherlands). Helium was used as carrier gas at a flow rate of 0.8 mL/min. 2 μl of sample was injected per run, or less if the signal exceeded the linear dynamic range of the instrument. Each sample was analyzed in duplicate. The sample was injected through the PTV injector in splitless mode. The initial oven temperature was 60°C and held constant for 0.5 min. Then the temperature was increased by 15°C/min to 150°C, and 4°C/min to 220°C. Then it was increased by 8°C/min to 300°C, and held constant for 7 min. After pyrolysis of the analytes, the enrichment of the hydrogen signal was monitored. Each analysis is corrected by within-run pulses of reference gas. The IRMS method included 5 pulses of a reference gas which was calibrated against the international certified standards Schimmelmann mixture A7 and USGS69. This was used to calibrate each run. The signal of the MS system was recorded from 500 s until 2400 s after start of the analysis. A calibration curve and control samples are measured during every series of samples. The linear dynamic range (upper and lower limit of quantification) are determined as a peak height between 0.1V and 25V. Analyses with results above or below these limits are repeated, with a higher or lower injection volume, or after dilution.

For AA enrichment analysis in meals, the lyophilized meals were hydrolyzed as described for ^13^C-enrichment analysis, and an aliquot of 1.35 mL of the same hydrolysate was prepared as described for the free AA in plasma for ^2^H enrichment.

## SUPPLEMENTARY TABLES

**Supplementary Table 1**. Macronutrient and amino acid composition of protein sources (milk, sorghum, black beans) and reference (free AA mixture) (CHO=carbohydrate).

| **Nutrient** | **Milk powder**  **(g/100g)** | **Sorghum**  **(g/100g)** | **Beans**  **(1^st^ batch)**  **(g/kg)** | **Beans**  **(2^nd^ batch)**  **(g/kg)** | **Free AA mixture**  **(g/100g)** |
| --- | --- | --- | --- | --- | --- |
| Protein | 34.9  N*6.38 | 9.3  N*5.4 | 20.3  N*5.4 | 22.2  N*5.4 |  |
| Fat | 0.8 | 4.3 | 1.7 | 1.9 |  |
| CHO | 52.5 | 72.5 | 61.5 | 57.9 |  |
| Ash | 8.1 | 1.6 | 4.5 | 3.3 |  |
| Water | 3.7 | 12.3 | 12.0 | 14.7 |  |
| Ala | 1.12 | 1.02 | 0.95 | 1.05 | 10.0 |
| Arg | 1.21 | 0.41 | 1.29 | 1.63 | 3.0 |
| Asn+Asp | 2.66 | 0.74 | 2.75 | 3.29 | 7.5 |
| Cys | 0.24 | 0.20 | 0.23 | 0.22 | 0.0 |
| Gln+Glu | 7.37 | 2.45 | 3.82 | 4.38 | 12.5 |
| Gly | 0.65 | 0.34 | 0.91 | 1.02 | 6.0 |
| His | 0.98 | 0.26 | 0.65 | 0.75 | 2.1 |
| Iso | 1.79 | 0.45 | 1.03 | 1.22 | 7.1 |
| Leu | 3.40 | 1.54 | 1.81 | 2.15 | 11.7 |
| Lys | 2.84 | 0.22 | 1.61 | 1.85 | 2.6 |
| Met | 0.83 | 0.21 | 0.26 | 0.28 | 1.7 |
| Phe | 1.67 | 0.58 | 1.28 | 1.53 | 8.9 |
| Pro | 3.51 | 0.94 | 0.79 | 0.89 | 4.4 |
| Ser | 1.88 | 0.49 | 1.34 | 1.57 | 5.3 |
| Thr | 1.51 | 0.35 | 0.97 | 1.06 | 6.3 |
| Trp | 0.42 | 0.12 | 0.24 | 0.24 | 0.0 |
| Tyr | 1.58 | 0.44 | 1.76 | 0.88 | 3.3 |
| Val | 2.18 | 0.56 | 1.19 | 1.37 | 7.7 |

**Supplementary Table 2**. Deuterium labelling protocol milk

| **Intake** | **Days** | **^2^H_2_O infusion (L)** | | | **Milk collection** |
| --- | --- | --- | --- | --- | --- |
|  |  | **6:00** | **12:00** | **17:30** |  |
| 1 | 1 | 0.6 | 0.6 | 0.6 |  |
| 2 | 2 | 0.6 | 0.6 | 0.6 |  |
| 3 | 3 | 0.6 | 0.6 | 0.6 |  |
|  | 4 | / | / | / | 5:00 and 16:00 |
|  | 5 | / | / | / | 5:00 and 16:00 |

**Supplementary Table 3**. Deuterium labelling protocol sorghum.

| **Intake** | **Days** | **%^2^H_2_O in total dilution** | **Total water/pot (mL)** | **^2^H_2_O/pot (mL)** | **H_2_O/pot (mL)** |
| --- | --- | --- | --- | --- | --- |
| 1 | 0 | 25 | 400 | 100 | 300 |
| 2 | 2 | 5 | 400 | 20 | 380 |
| 3 | 4 | 5 | 400 | 20 | 380 |
| 4 | 6 | 5 | 400 | 20 | 380 |
| 5 | 8 | 5 | 400 | 20 | 380 |

**Supplementary Table 4**. Deuterium labelling protocol first batch black beans.

| **Intake** | **Days** | **%^2^H_2_O in total dilution** | **Total water/pot (mL)** | **^2^H_2_O/pot (mL)** | **H_2_O/pot (mL)** |
| --- | --- | --- | --- | --- | --- |
| 1 | 0 | 25 | 200 | 50 | 150 |
| 2 | 2 | 5 | 200 | 10 | 190 |
| 3 | 4 | 5 | 200 | 10 | 190 |
| 4 | 6 | 5 | 200 | 10 | 190 |
| 5 | 8 | 5 | 200 | 10 | 190 |

**Supplementary Table 5**. Deuterium labelling protocol second batch black beans.

| **Intake** | **Days** | **%^2^H_2_O in total dilution** | **Total water/pot (mL)** | **^2^H_2_O/pot (mL)** | **H_2_O/pot (mL)** |
| --- | --- | --- | --- | --- | --- |
| 1 | 0 | 25 | 200 | 50 | 150 |
| 2 | 2 | 5 | 300 | 15 | 285 |
| 3 | 4 | 5 | 300 | 15 | 285 |
| 4 | 6 | 5 | 300 | 15 | 285 |
| 5 | 8 | 5 | 300 | 15 | 285 |

**Supplementary Table 6**. Estimated Marginal Means for lysine and threonine in full steady state plasma-to-meal ratios and their age effect

|  | **Milk** | | **Sorghum** | | **Black beans** | |
| --- | --- | --- | --- | --- | --- | --- |
|  | Young | Old | Young | Old | Young | Old |
| **Lys** | 0.77 | 0.72 | 0.48 | 0.40 | 0.67 | 0.67 |
| **Thr** | 0.88 | 0.84 | 0.37 | 0.28 | 0.75 | 0.75 |
| **SE** | 0.03 | 0.03 | 0.03 | 0.03 | 0.03 | 0.03 |
| **P** | 0.270 | | 0.040 | | 0.976 | |

**Supplementary Table 7**. Estimated Marginal Means for lysine and threonine in AUC plasma-to-meal ratios and their age effect

|  | **Milk** | | **Sorghum** | | **Black beans** | |
| --- | --- | --- | --- | --- | --- | --- |
|  | Young | Old | Young | Old | Young | Old |
| **Lys** | 0.74 | 0.75 | 0.40 | 0.34 | 0.63 | 0.64 |
| **Thr** | 0.84 | 0.86 | 0.28 | 0.22 | 0.68 | 0.67 |
| **SE** | 0.03 | 0.03 | 0.03 | 0.03 | 0.03 | 0.03 |
| **P** | 0.674 | | 0.150 | | 0.863 | |

## SUPPLEMENTARY FIGURES

**Supplementary figure 1**. Sorghum and bean puree

**Supplementary figure 2**. Average with one sided standard deviation ^2^H enrichment for young adults given milk, sorghum, black beans and for older adults given milk, sorghum, black beans

**Supplementary Figure 3**. Average with one sided standard deviation ^13^C enrichment for young adults given milk, sorghum, black beans and for older adults given milk, sorghum, black beans

**Supplementary Figure 4**. Isotope plasma-to-meal ratio (mean with standard deviation) based on full steady state (5.5-8.0 hours) for indispensable amino acids (AA, His: histidine, Ile: isoleucine, Leu: leucine, Lys: lysine, Met: methionine, Phe: phenylalanine, Thr: threonine, Val: valine) separated for young and older adults for milk (A; young n=10; old n=9, for threonine young n=8), sorghum (B; young n=9; old n=10) and black beans (C; young and old n=10). Only for Lys and Thr the isotope plasma-to-meal ratio is a measure of digestibility and can be compared between age groups.

**Supplementary Figure 5**. Isotope plasma-to-meal ratio (mean with standard deviation), for indispensable amino acids (AA, His: histidine, Ile: isoleucine, Leu: leucine, Lys: lysine, Met: methionine, Phe: phenylalanine, Thr: threonine, Val: valine) separated for young and older adults for milk (A; young n=10; old n=9, for threonine young n=8), sorghum (B; young n=9; old n=10) and black beans (C; young and old n=10). Only for Lys and Thr the isotope plasma-to-meal ratio is a measure of digestibility and can be compared between age groups.

**Supplementary Figure 6**. Isotope plasma-to-meal ratio (mean with standard deviation) based on AUC, for indispensable amino acids (AA, His: histidine, Ile: isoleucine, Leu: leucine, Lys: lysine, Met: methionine, Phe: phenylalanine, Thr: threonine, Val: valine) separated for young and older adults for milk (A; young n=10; old n=9, for threonine young n=8), sorghum (B; young n=9; old n=10) and black beans (C; young and old n=10). Only for Lys and Thr the isotope plasma-to-meal ratio is a measure of digestibility and can be compared between age groups.

**REFERENCES**

(1) Kampman-van de Hoek E, Sakkas P, Gerrits WJ, van den Borne JJ, van der Peet-Schwering CM, Jansman AJ. Induced lung inflammation and dietary protein supply affect nitrogen retention and amino acid metabolism in growing pigs. Br J Nutr. 2015;113(3):414-25.
(2) Vlaardingerbroek H, Vermeulen MJ, Rook D, van den Akker CH, Dorst K, Wattimena JL, et al. Safety and efficacy of early parenteral lipid and high-dose amino acid administration to very low birth weight infants. J Pediatr. 2013;163(3):638-44.e1-5.
